# Supplementary material for: The ω3 scaling of the vibrational density of states in quasi-2D nanoconfined solids
Source: Nat Commun. 2022 Jun 25;13:3649. doi: 10.1038/s41467-022-31349-6 (PMC9233700; doi:10.1038/s41467-022-31349-6)
Supplement: Supplementary file 1 — Supplementary Information [file 41467_2022_31349_MOESM1_ESM.pdf]

Supplementary Information

The ω^3 scaling of the vibrational density of states in quasi-2D nanoconfined solids

Yuanxi Yu, Chenxing Yang, Matteo Baggioli*, Anthony E. Phillips, Alessio Zaccone, Lei Zhang, Ryoichi Kajimoto, Mitsutaka Nakamura, Dehong Yu, Liang Hong*

Supplementary Figures

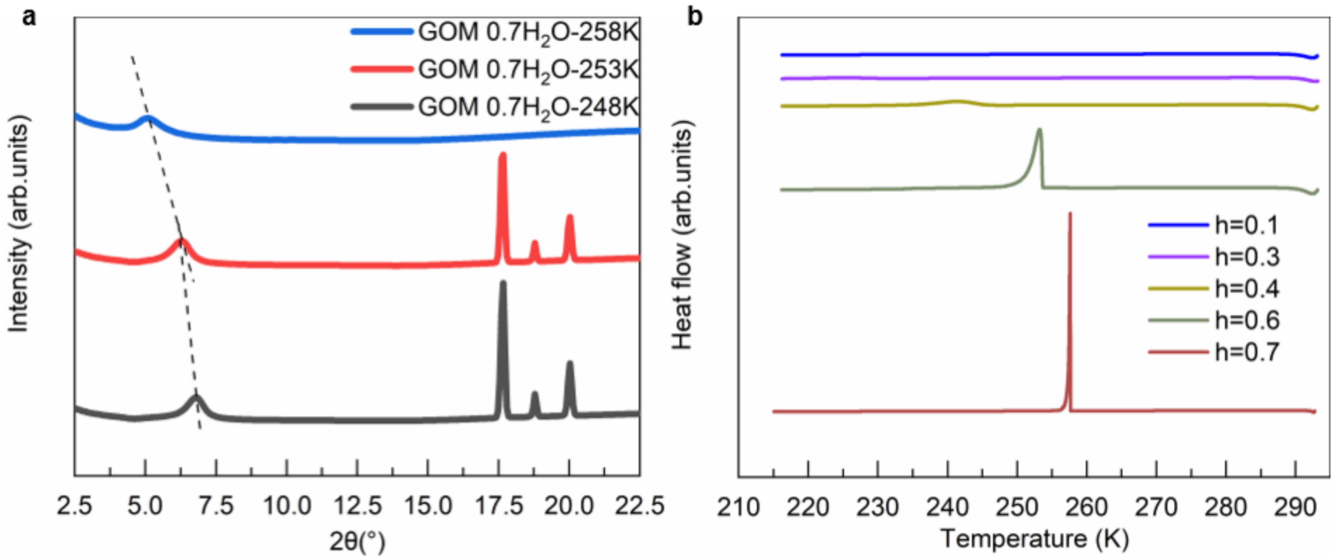

Supplementary Fig. 1. Additional structural and thermodynamic characterization of GOM samples. (a) The SAXS data of GOM with $h = 0.7$ at different temperatures, showing the characteristic peaks of the GOM layer spacing shift with the formation of ice. **(b)** The DSC result during the cooling process for the GOM samples with different hydration levels.

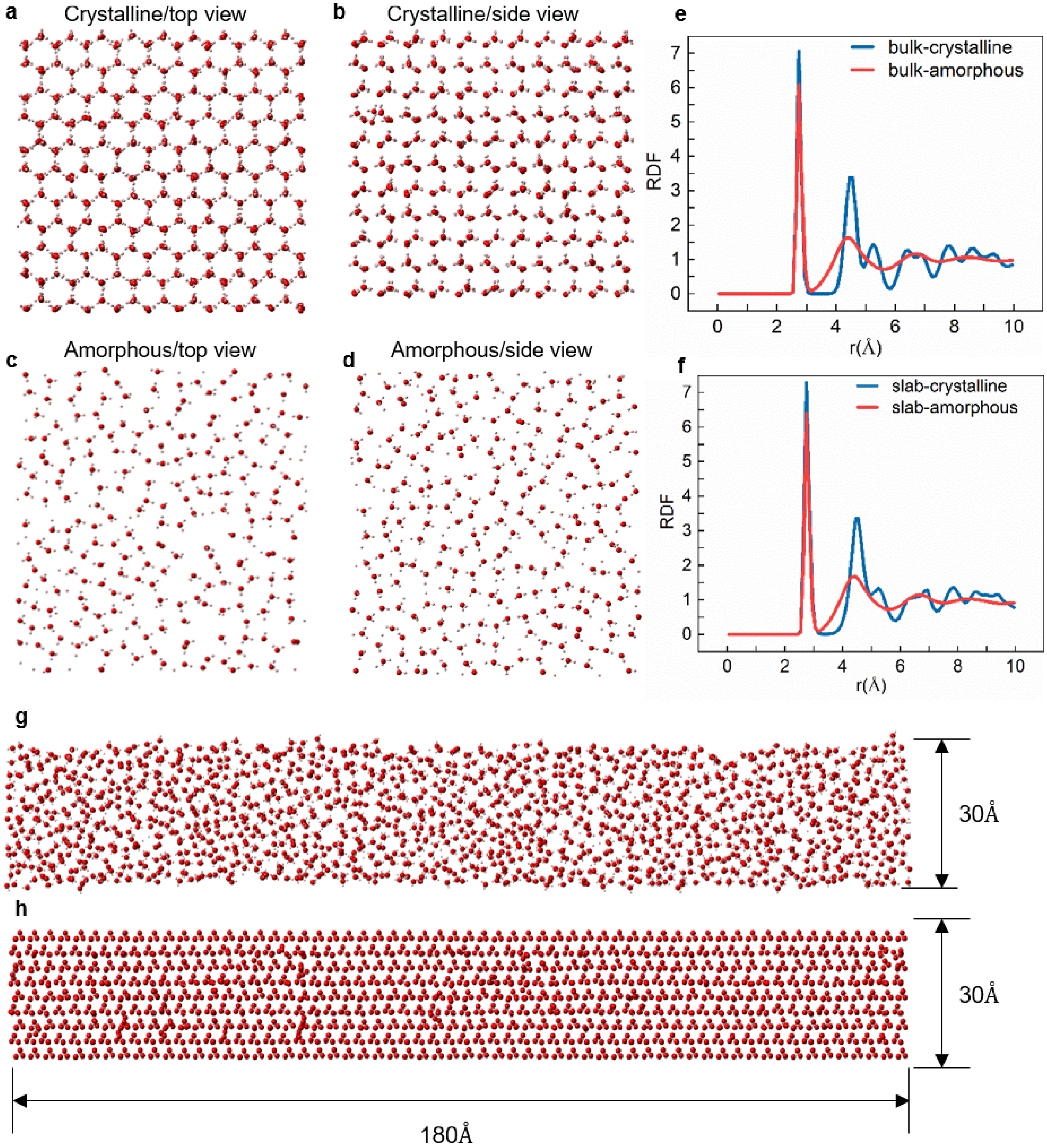

Supplementary Fig. 2. Snapshots from different points of view of the crystalline and amorphous ice structures used in the MD simulations. **(a)** Top view of crystalline ice. **(b)** Side view of crystalline ice. **(c)** Top view of amorphous ice. **(d)** Side view of amorphous ice. The radial distribution function for the bulk ice **(e)** and slab ice samples **(f)**. The snapshots of amorphous **(g)** and crystalline **(h)** ice slab samples with size 180Å and confinement length along the z direction 30Å. For visual purposes, only few layers are kept in all the snapshots.

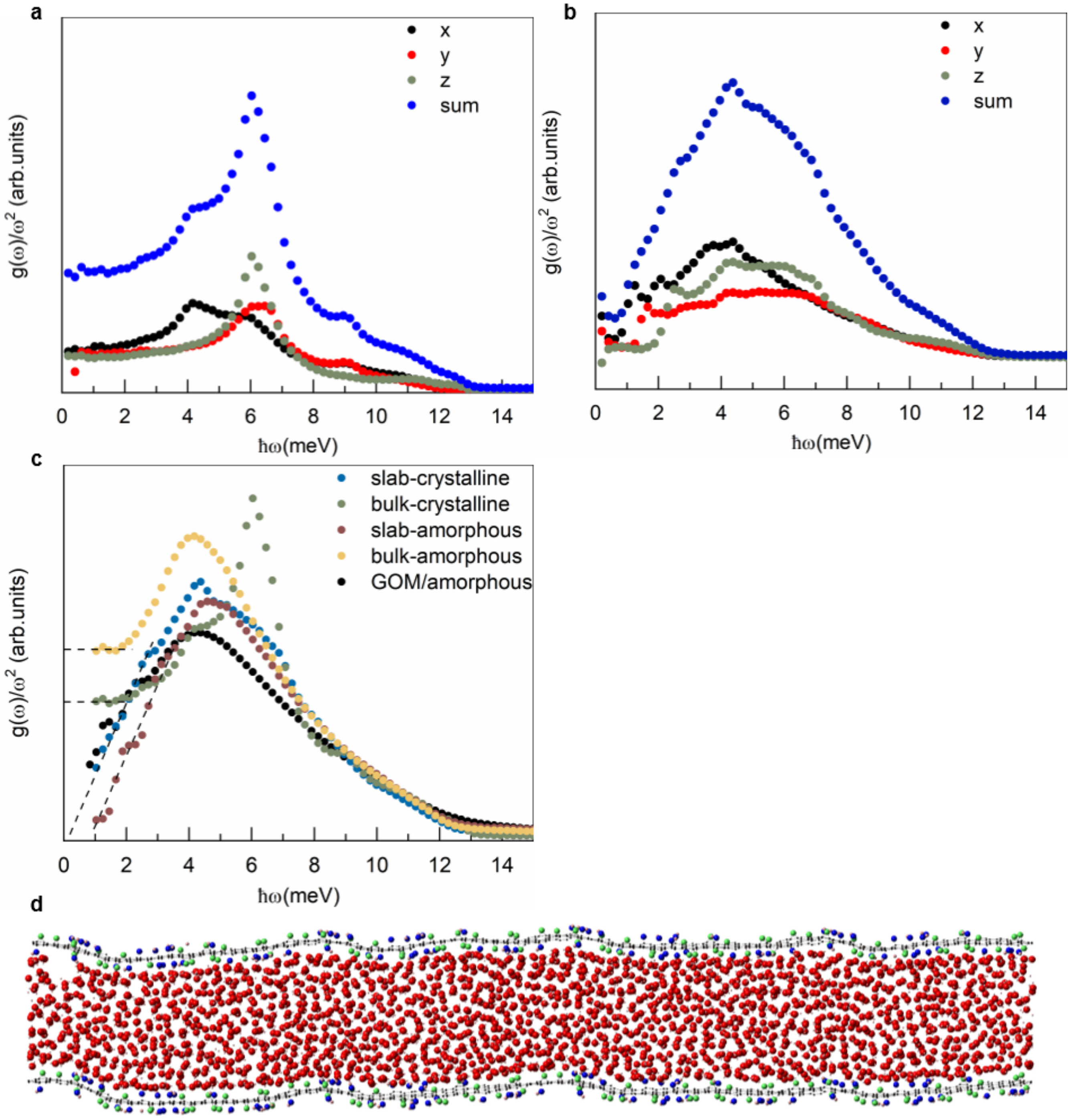

Supplementary Fig. 3. **(a)** The VDOS of bulk ice calculated from the velocity auto-correlation function along different axes. **(b)** The VDOS of amorphous ice calculated from the velocity auto-correlation function along different axes. **(c)** The normalized VDOS in function of frequency obtained from the MD simulations for slab crystalline ice, bulk crystalline ice, slab amorphous ice,bulk amorphous ice and amorphous ice confined in GOM. **(d)** The actual structure of GOM confined amorphous ice in simulations. The epoxy group on the GOM is marked by a green oxygen atom and the hydroxyl group by a blue one.

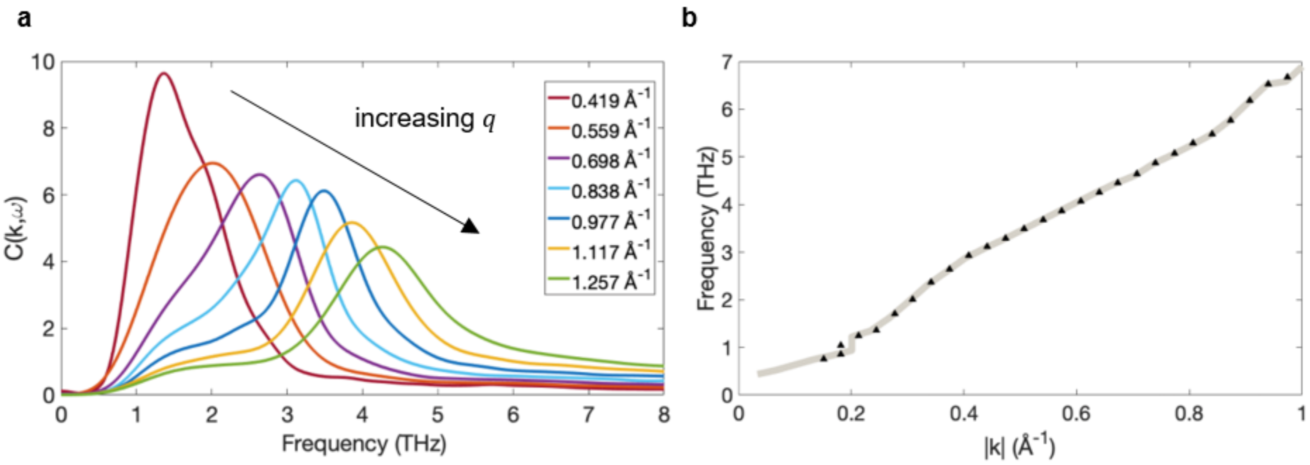

Supplementary Fig. 4. (a) The longitudinal current correlations spectrum at different k_x with fixed $k_z = 0.07\text{Å}^{-1}$. The arrow indicates the motion of the intensity peak upon moving the wavevector k_x. **(b)** The dispersion relation as a function of $|k|$. The grey line represents the dispersion relation with wavevector parallel to the xy plane. The black triangles represent the dispersion relation with wavevector $k_z = 0.07\text{Å}^{-1}$.

Supplementary Note

Additional experimental data. In Supplementary Fig.1(a), the characteristic peak of the GOM sample layer spacing is shifting towards larger angles with the temperature decreasing and the formation of ice. This shows that as the temperature drops, the water confined in the GOM layers migrates to some voids or to the surface of GOM to form bulk crystalline ice. Therefore, the GOM layer spacing decreases significantly. This mechanism is known as the ice segregation process.(1; 2; 3). The DSC measurements for samples with different hydration levels are reported in Supplementary Fig.1(b). No first-order transition is observed at $h = 0.3$ or below, but it appears at higher hydration levels, $h = 0.4$ and above. As a result, we can note that in the samples with water content of 0.3 and below, the crystalline ice cannot be formed, in agreement with our SAXS results in Fig.3(c) of the main text. The ratio of the bulk ice and amorphous ice is calculated using the latent heat of the first order phase transition of water and reported in panel (d) of Fig.3 in the main text.

Details of the simulations setup. The snapshots of the amorphous and crystalline ice samples used in the MD simulations are shown in Supplementary Fig.2. In the crystalline ice setup, the water molecules are arranged into an ordered hexagonal lattice, which can be seen from different points of view in Supplementary Figs.2(a) and (b). On the contrary, the highly disorder structure of the amorphous ice sample can be seen in Supplementary Figs.2(c) and (d). In order to confirm the crystalline/amorphous nature of the different samples, we show in Supplementary Figs.2(e) and (f) the corresponding radial distribution function (RDF). The RDF of the crystalline ice samples shows sharp peaks (indicating the presence of long range order) which are absent in the amorphous counterparts. The same contrast can be observed in the overall snapshots of amorphous/crystalline slab samples in Supplementary Figs.2(g) and (h).

As a reference for our simulations under confinement, we have computed the velocity auto-correlation function in different coaxial directions for the crystalline bulk ice sample. The results are shown in Supplementary Figs.3(a) and (b) where a sharp peak in the VDOS is observed around ≈ 6.2 meV. Using the analysis in the various directions, we can conclude that the sharp peak in the VDOS of bulk crystalline ice in Supplementary Fig.3(d) comes from the vibrations perperdicular to the [0001] basal plane of the hexagonal ice structure. In both the amorphous and slab-crystalline samples, this peak becomes broader and moves slightly to lower frequencies, ≈ 4.5 meV. This effect can be explained by an increase of the linewidth of the corresponding excitation. In the case of the amorphous systems, this broadening of the linewidth naturally arises because of structural disorder. In the case of the slab-crystalline sample, the spatial confinement along the z direction reduces the constructive interference and thus decreases the peak intensity. Regarding the nature of this peak, there is yet no consensus in the literature. Some works identify this excess mode as a genuine boson peak(4; 5), others(6; 7; 8) attribute this excess to a specific optical mode of the crystalline ice structure.

In the main text, the simulation analysis is conducted without including the dynamics of the GOM. The reasons behind this choice are the following: first, the slab simulation (without GOM) is easier to set up for both crystalline and amorphous phases, and thus one can make a fair comparison of the two systems under the same confinement. In contrast, GOM has a rather rough and curvy surface (see Supplementary Fig.3(d)) which renders the simulations for the confined crystalline ice difficult. Secondly, precise control of the thickness of the sample is needed in order to test the theoretical derivation for the crossover scale between the ω^3 and ω^2 scalings. Again, the rough and curvy surface of GOM prevents such an analysis. To make sure that the absence of the GOM in the simulations does not affect, at least qualitatively, our main results we have conducted simulations including the GOM dynamics as well. The comparison of the VDOS for bulk crystalline ice, slab crystalline ice, bulk amorphous ice, slab amorphous ice and amorphous ice confined in GOM is shown in Supplementary Fig.3(c). The VDOS of the GOM-sandwiched amorphous ice is in qualitative agreement with that obtained from the slab setup without GOM. We therefore conclude that the absence of GOM in the simulations does not affect our conclusions.

Failure of the hard-wall boundary conditions. Let us consider a simple wave equation in a three-dimensional box of size $L_x \times L_y \times L_z$. For simplicity, and importantly not for any fundamental physical reason, such an equation is usually solved by assuming the so-called smooth hard-wall boundary conditions, namely that the displacement field vanishes on all the edges of the box. Under these assumptions, and neglecting the time dynamics of the wave-equation since irrelevant for our purposes, the solution is simply:

$$\phi(x,y,z) = \sin(k_x x)\sin(k_y y)\sin(k_z z) \qquad \text{(Supplementary 1)}$$

supplemented by the following conditions:

$$\sin(k_x L_x) = \sin(k_y L_y) = \sin(k_z L_z) = 0 \qquad \text{(Supplementary 2)}$$

which are immediately solved by:

$$k_x = i\frac{\pi}{L_x}, \qquad k_y = j\frac{\pi}{L_y}, \qquad k_z = k\frac{\pi}{L_z}, \qquad \text{(Supplementary 3)}$$

with i, j, k integers.
The hard-wall boundary conditions have therefore two striking consequences. (I) the wavevector is discrete and (II) there is a minimum wavevector.
Going back to our experimental sample, there we can take safely $L_x, L_y \to \infty$. Nevertheless, this would still imply that $|k| = \sqrt{k_x^2 + k_y^2 + k_z^2}$ is bounded from below by:

$$|k| > \frac{\pi}{L} \qquad \text{(Supplementary 4)}$$

where $L = L_z$ as in the main text.
If this argument were true, our theoretical explanation presented in the main text would be clearly invalid (see for example (9), where periodic boundary conditions were instead used in the simulations). Nevertheless, as we showed directly here using MD simulations, this is not the case and the smooth hard-wall boundary conditions do not apply to our system. As an immediate consequence, (I) our wavevector is not discrete and (II) our wavevector is not limited from below by π/L.

In Supplementary Fig. 4, we calculated the longitudinal current correlation function and dispersion relation in the supercooled liquid slab. The wavevector along the z direction k_z is fixed at ~ 0.07 Å^{-1}, which corresponds to $\frac{\pi}{2L}$, much below the minimal value allowed by hard-wall bcs. In Supplementary Fig.4(a), we can clearly see that the peak position shifts to higher frequency with increasing $|k|$. This results suggest that a well-defined propagating mode exists also for values of k_z which are not allowed by the hard-wall bcs. In addition, we compare the dispersion relation for wavevector $k_z = 0.07$Å^{-1} with that for wavevector parallel to the xy plane in Supplementary Fig.4 (b). From there, we observe that two dispersion relations strongly overlap, meaning the dispersion relation is only related to the norm of the wavevector $|k|$.

Supplementary References

1. Gutiérrez, M. C., Ferrer, M. L. & del Monte, F. Ice-templated materials: Sophisticated structures exhibiting enhanced functionalities obtained after unidirectional freezing and ice-segregation-induced self-assembly. Chem. Mater. **20**, 634–648 (2008).
2. Murton, J. B., Peterson, R. & Ozouf, J.-C. Bedrock fracture by ice segregation in cold regions. Science **314**, 1127–1129 (2006).
3. Öberg, K. I., Fayolle, E. C., Cuppen, H. M., van Dishoeck, E. F. & Linnartz, H. Quantification of segregation dynamics in ice mixtures. Astron. & Astrophys. **505**, 183–194 (2009).
4. Tse, J. et al. Origin of low-frequency local vibrational modes in high density amorphous ice. Phys. Rev. Lett. **85**, 3185 (2000).
5. Li, J. & Kolesnikov, A. The first observation of the boson peak from water vapour deposited amorphous ice. Phys. B: Condens. Matter **316**, 493–496 (2002).
6. Schober, H. et al. Crystal-like high frequency phonons in the amorphous phases of solid water. Phys. review letters **85**, 4100 (2000).
7. Koza, M. M. Vibrational dynamics of amorphous ice structures studied by high-resolution neutron spectroscopy. Phys. Rev. B **78**, 064303 (2008).
8. Koza, M., Schober, H., Parker, S. & Peters, J. Vibrational dynamics and phonon dispersion of polycrystalline ice xii and of high-density amorphous ice. Phys. Rev. B **77**, 104306 (2008).
9. Yang, J., Li, Y.-W. & Ciamarra, M. P. Long-wavelength fluctuations and dimensionality crossover in confined liquids. Phys. Rev. Res. **3**, 033172 (2021).
